# Levetiracetam for Pediatric Posttraumatic Seizure Prophylaxis

**DOI:** 10.15844/pedneurbriefs-30-3-1

**Published:** 2016-03

**Authors:** Dragos A. Nita, Cecil D. Hahn

**Affiliations:** 1 Division of Neurology, The Hospital for Sick Children and Department of Paediatrics, University of Toronto, Toronto, ON, Canada; 2 Program in Neuroscience and Mental Health, The Hospital for Sick Children Research Institute, Toronto, ON, Canada

**Keywords:** PICU, Antiepileptics, Epilepsy

## Abstract

Investigators from Nationwide Children's Hospital performed an observational cohort study of early post-traumatic seizures (EPTS) among 34 children with moderate to severe traumatic brain injury (TBI) who received levetiracetam (LEV) prophylaxis following admission to their pediatric intensive care unit.

Investigators from Nationwide Children's Hospital performed an observational cohort study of early post-traumatic seizures (EPTS) among 34 children with moderate to severe traumatic brain injury (TBI) who received levetiracetam (LEV) prophylaxis following admission to their pediatric intensive care unit. EPTS were defined as clinical seizures occurring within seven days from the brain injury. The authors found that 6/34 (17%) children developed EPTS despite LEV prophylaxis. The authors conclude that EPTS remain common despite LEV prophylaxis, and that young children and those suffering abusive head trauma are at particularly high risk. [1]

COMMENTARY. EPTS are common following moderate to severe TBI, and are associated with worse outcome [2]. These seizures may contribute to secondary brain injury through a variety of mechanisms, including regional hypoxia-ischemia due to the increased metabolic demands of seizures, glutamate-mediated excitotoxicity, and increased intracranial pressure. Current guidelines for the management of severe TBI published by the American Academy of Neurology [3] and the Brain Trauma Foundation [4] recommend acute seizure prophylaxis during the first seven days after TBI. Prophylactic phenytoin has been shown to reduce the prevalence of EPTS in both adults and children [5, 6]. Phenobarbital, carbamazepine and valproic acid have not been as extensively investigated, but given their side-effect profiles and pharmacodynamic properties, there is no clear advantage to using these agents over phenytoin [7]. On the other hand, LEV has become a popular choice for EPTS prophylaxis in many centers, prompted by its favorable side-effect profile compared to phenytoin [8].

Despite the growing popularity of LEV for EPTS prophylaxis in children with TBI [8], evidence for its efficacy remains scant. Hence, this study is an important contribution. The authors conclude that LEV may be less effective than phenytoin in preventing EPTS because the observed prevalence of EPTS (17%) was higher than previously reported with phenytoin prophylaxis (2-15%). However, this was not a comparative study, therefore other clinical factors may have accounted for the higher prevalence of EPTS observed in this cohort. Nevertheless, these findings highlight the need for a prospective randomized controlled trial to compare the safety and efficacy of LEV vs. phenytoin for the prevention of EPTS. Ideally, this study should apply continuous EEG monitoring to identify children with seizures because of the high prevalence of subclinical seizures known to occur in this population [9].
